# A Flexible Transoral Robot Towards COVID-19 Swab Sampling

**DOI:** 10.3389/frobt.2021.612167

**Published:** 2021-04-12

**Authors:** Changsheng Li, Xiaoyi Gu, Xiao Xiao, Chwee Ming Lim, Xingguang Duan, Hongliang Ren

**Affiliations:** ^1^ School of Mechatronical Engineering, Beijing Institute of Technology, Beijing, China; ^2^ Beijing Advanced Innovation Center for Intelligent Robots and Systems, Beijing Institute of Technology, Beijing, China; ^3^ Department of Biomedical Engineering, National University of Singapore, Singapore, Singapore; ^4^ NUS (Suzhou) Research Institute (NUSRI), Suzhou, China; ^5^ Department of Electrical and Electronic Engineering, Southern University of Science and Technology, Shenzhen, China; ^6^ Department of Otolaryngology-Head and Neck Surgery, Singapore General Hospital, Singapore, Singapore; ^7^ Duke-NUS Graduate Medical School, Singapore, Singapore; ^8^ Department of Electronic Engineering, The Chinese University of Hong Kong (CUHK), Hong Kong, China

**Keywords:** COVID-19, swab sampling, transoral robot, Flexible robot, sampling robot, Flexible parallel mechanism, surgical robotics, medical robotics

## Abstract

There are high risks of infection for surgeons during the face-to-face COVID-19 swab sampling due to the novel coronavirus’s infectivity. To address this issue, we propose a flexible transoral robot with a teleoperated configuration for swab sampling. The robot comprises a flexible manipulator, an endoscope with a monitor, and a master device. A 3-prismatic-universal (3-PU) flexible parallel mechanism with 3 degrees of freedom (DOF) is used to realize the manipulator’s movements. The flexibility of the manipulator improves the safety of testees. Besides, the master device is similar to the manipulator in structure. It is easy to use for operators. Under the guidance of the vision from the endoscope, the surgeon can operate the master device to control the swab’s motion attached to the manipulator for sampling. In this paper, the robotic system, the workspace, and the operation procedure are described in detail. The tongue depressor, which is used to prevent the tongue’s interference during the sampling, is also tested. The accuracy of the manipulator under visual guidance is validated intuitively. Finally, the experiment on a human phantom is conducted to demonstrate the feasibility of the robot preliminarily.

## 1 Introduction

Coronavirus disease 2019 (COVID-19) transmitted through respiratory droplets is spreading rapidly (Chaolin et al., 2020). The widely used diagnose method is the oropharyngeal-swab (OP-swab) sampling (Chen et al., 2020). This sampling is suggested to be performed by a healthcare professional, while other possible approaches such as nasal mid-turbinate swab could be self-collection under supervised (Kim et al., 2020). The healthcare workers who perform swab sampling face high infection risks caused by the testees’ aerosol during the sampling, as close person to person contact is the main way for the transmission of the virus (Jin et al., 2020). Besides, the sample’s quality depends on the operators’ skills, which is inconsistent and may cause misdiagnosis (Li et al., 2020b). As highlighted in (Yang et al., 2020), one of the effective solutions is using the teleoperated robot that can keep the operators safe by avoiding close contact with the testees (Navid et al., 2021; Xiao et al., 2021).

Accurate swab delivery to the target region with reasonable force is essential for the teleoperated robot-assisted swab sampling (Gu et al., 2019; Hosokawa-Muto et al., 2019). Enough dexterity for the robot with at least three degrees of freedom is required to ensure that the robot can reach the target region and to wipe through the mouth. The typical configurations are two bending DOFs with one translational DOF, or one bending DOF, one rotational DOF with one translational DOF. The workspace of the robot needs to cover the area of throat with a maximum size of 23 mm × 20 mm. The anteroposterior length of the tongue is less than 75 mm (Miguel et al., 2007). As the applied force determined the keenly feel of the testees, the robot is recommended to be compliant enough through there is no quantitation standard currently. Teleoperation is usually carried out under visual guidance. In this situation, the operator stands by the master console and teleoperates the slave robot. During the operation, the swab sampling’s safety is an indispensable factor that can be achieved by a flexible mechanism or control algorithm (Park, 2007; Ren et al., 2013). As the potential irritation of the swab sampling for the testee, the operator should consider how to prevent the tongue to block the view (Sahin and Dogan, 2019). Besides, the sterilizability of the robot to prevent cross-infection should also be considered (Porter et al., 2020). Currently, the swab sampling robots are under development around the world (Wang et al., 2020), with some of them being preliminarily applied in the clinic (Li et al., 2020b). However, due to the research activities’ limitation, most of the robots are still incomplete with a simple structure.

This paper proposes a flexible transoral robot towards COVID-19 swab sampling, aiming to reduce the risks of infection for the healthcare workers during the sampling. The contributions can be detailed as follows:

• A robotic system with a flexible mechanism is proposed, achieving a dexterity sampling with flexibility.
• The tongue depressor is designed for safe operation.
• The experiments, including the parameter and the performance tests of the robot, are conducted.

The rest of this paper is organized as follows. In Section 2, the robotic system with the workspace and the operation procedure is introduced in detail. The experimental evaluation is demonstrated. Section 3 presents the results of the experiments. Finally, the discussion is presented in Section 4.

## 2 Materials and Methods

### 2.1 System Description

#### 2.1.1 Robotic System

**FIGURE 1:**
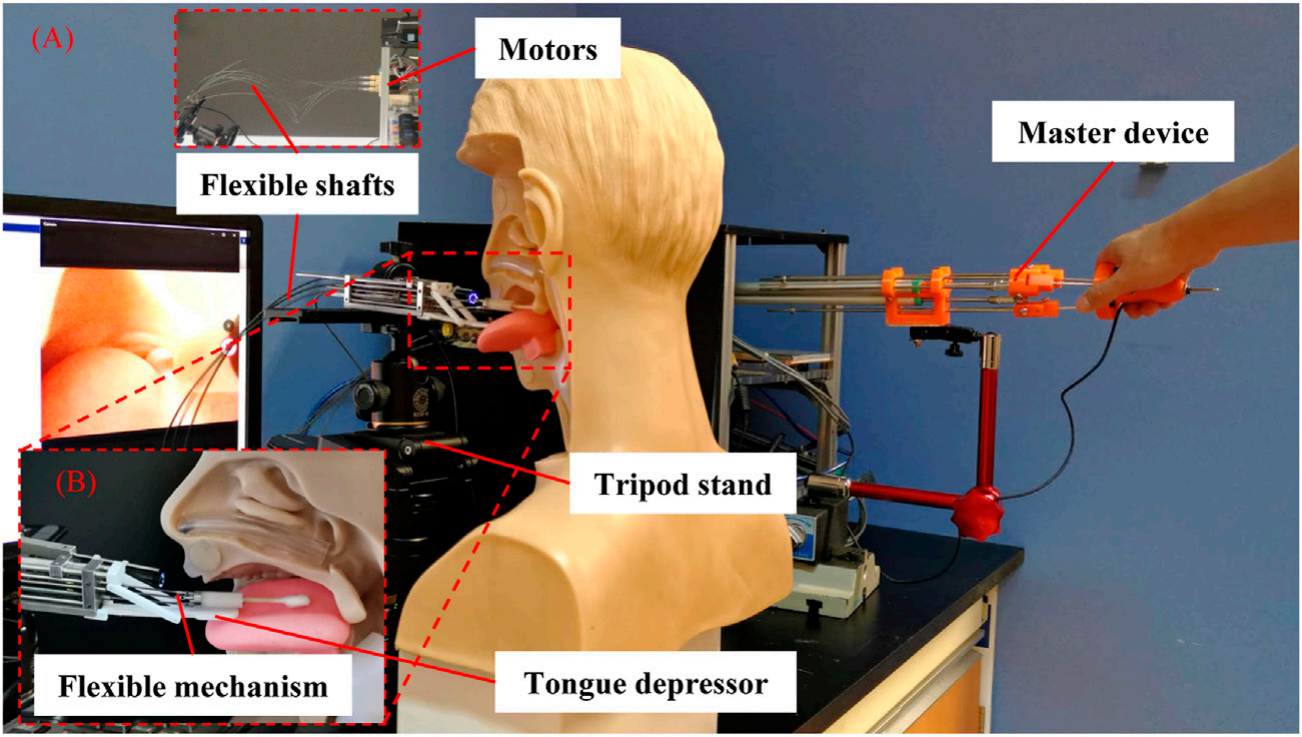
Flexible transoral robot. **(A)** The robotic system; **(B)** The flexible manipulator.

The robotic system consists of a flexible manipulator with a tongue depressor mounted on a tripod stand, an endoscope (Diameter: 5.5 mm, Resolution: 1280 × 720, HL-5520, Hlisen Inc.) with a monitor for the guidance of the operation, and a master console with a master device and a controller (Figure 1A). To achieve the sampling, the surgeon stands by the master console and operates the master device to control the manipulator’s motion under the guidance of vision obtained from the endoscope.

As shown in Figure 1B, the flexible parallel mechanism is designed as the manipulator’s terminal. The super-elastic Ni-Ti rods (Diameter: 0.78 mm, Young’s modulus: 20 Gpa, Suzuki-Sumiden Wire Products Co., Ltd.) with universal joints (MAASS-1.0, Misumi South East Asia Pte Ltd.) are used as the chains to construct a 3-prismatic-universal (3-PU) mechanism. The chains are driven by motors placed far away from the manipulator via thread rods and flexible shafts. To be specific, the motors are connected to the thread rods via flexible shafts. The thread rods are used to drive the motion of the flexible mechanism by translating the rotational motion of the flexible shafts to the translational motion of the Ni-Ti rods. The manipulator diameter is 8 mm. There are three DOFs, including two bending DOFs and one translational DOF. More details about this flexible parallel mechanism including the kinematic, stiffness analysis, evaluation, and master-slave operative performance can be found in our previous work (Li et al., 2019a; Li et al., 2019b). In brief, the manipulator can be operated stably and accurately with proper flexibility by combining the parallel mechanism and the flexible mechanism. The swab stick for sampling can be attached or removed from the manipulator’s terminal via its mechanical interface. As the swab stick is in contact with the tissue of testee during the sampling, the compliance of the manipulator is helpful to alleviate the testee’ pain and increase their safety. In our design, it is achieved via the Ni-Ti rods with super-elastic characteristic. The level of the compliance is determined by the stiffness of the rods, which is related with the sizes of their diameters.

During the sampling, there is a possibility that the swab stick would be disturbed by the spontaneous action of the tongue caused by the irritation of the throat, leading to the failure of the sampling or even additional injuries. So the tongue depressor composed by a linkage mechanism is designed to restrict the motion of the tongue. The tongue depressor can be controlled to rotate in real-time during the sampling and reset to the initial state when the manipulator inserts or retreats from the oral cavity.

The master device is mainly composed of a parallel mechanism with three prismatic-revolute-spherical (3-PRS) chains, similar to the flexible manipulator in structure. This kind of design plays an active role in decreasing the learning curve for less experienced surgeons (Li et al., 2020a). Three displacement sensors (LPZ-200, Fiaye Electric Co., Ltd.) are attached to the chains to detect the pose of the master device. Another displacement sensor (LPZ-20, Fiaye Electric Co., Ltd.) is connected to the master device terminal to control the motion of the tongue depressor. Three DOFs are achieved, including two bending DOFs and one translational DOF. The flexible manipulator’s movement can be amplified by five times, allowing the operator’s delicate operation. The time delay of the master-slave control has been tested in our previous work (Li et al., 2019a; Li et al., 2019b).

The personal computer (PC) with SimLab boards (Zeltom LLC.) running Matlab/Simulink programs is used as a controller. The input pose signals of the master device and the tongue depressor’s control signals are obtained from the displacement sensors. The output signals are used to control the motion of the flexible manipulator via flexible shafts (Diameter: 1.7 mm, Hagitec Co., Ltd.) and screws (Diameter: 3 mm, lead: 0.5 mm) driven by direct current (DC) motors (RE13, Maxon motor Inc.).

#### 2.1.2 Workspace Characterization

**FIGURE 2:**
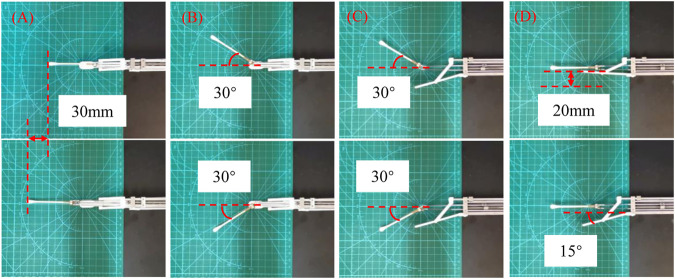
Workspace demonstration of the robot. **(A)** The maximum displacement; **(B)** The maximum bending angle in top view; **(C)** The maximum bending angle in side view; **(D)** The motion range of the tongue depressor.

The workspace of the robot is evaluated by driving the manipulator to its limit positions, which is shown in Figure 2. The maximum displacement of the manipulator is 30 mm (Figure 2A), and the maximum bending angles of the manipulator in its top view and its side view are both 30° (Figures 2B,C). The displacement and the manipulator’s bending angle are determined by both of the mechanical structure of the manipulator and the master device. The results show that if the swab stick’s length is 60 mm, the workspace of the robot covers a cylinder space with a diameter of 60 mm and a height of 30 mm, which is large enough for the swab sampling compared with the size of the throat. As the manipulator can be placed close the throat before sampling, the translational distance of the manipulator is allowed to be shorter than the length of the anteroposterior length of the tongue. The tongue depressor’s maximum moving distance is 20 mm, with a bending angle of 15° (Figure 2D).

#### 2.1.3 Operation Procedure

The operation procedure is described as follows. Before the operation, the sterilized wraps are attached to the flexible manipulator, and the plastic wrap with high light transmittance are covered on the endoscope to prevent the possibility of cross-infection. After that, the robotic system is set to the initial state. The operator stands by the master console in the isolated condition. The testee faces to the mechanical design and opens the mouth. When the tongue’s tip props up the tongue depressor, the operator begins to operate the flexible manipulator. The tongue depressor is controlled to hold down the tongue and allows the swab stick to reach the throat. The secret can be collected by quickly wiping the palatal arch, pharynx, and tonsil. After the operation, the swab stick and the tongue are retrieved.

### 2.2 Experimental Evaluation

Three experiments were conducted to test the pressure of the tongue depressor, the accuracy of the manipulator under visual guidance, and the robotic system’s performance on the human phantom (see videos in Supplementary Materials).

#### 2.2.1 Pressure Tests of the Tongue Depressor

**FIGURE 3:**
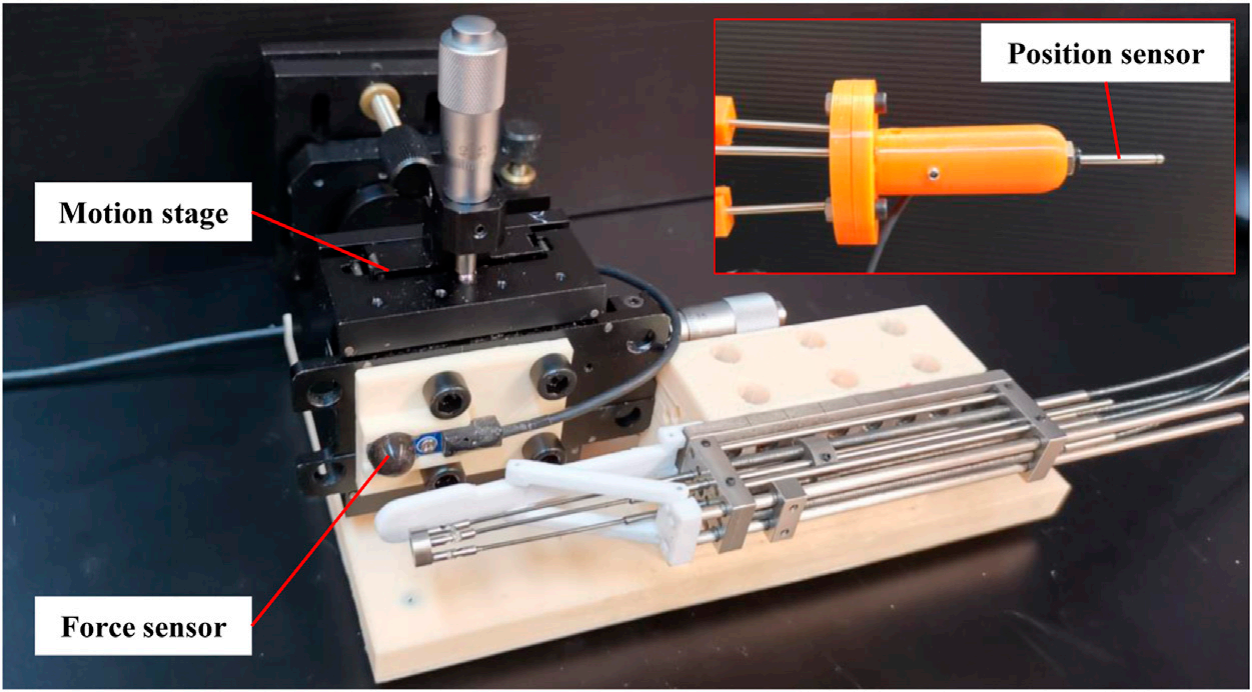
Setup for the pressure tests of the tongue depressor.

The pressure on the tongue provided by the tongue depressor should be larger than the forward force of tongue to prevent the tongue’s disturbance. Nevertheless, overload pressure may lead to the increased risks of tongue injuries. So the maximum pressure of the tongue depressor is tested in this section. As shown in Figure 3, the force sensor (Resolution: 2.5 mN, OMD-10-SE-10N, Optoforce Ltd.) and the manipulator were attached to the motion stage via the 3D printed support. The tongue depressor was driven to rotate under the control of the position sensor attached to the master device. The force sensor detected the pressure from the terminal of the tongue depressor. This process was repeated ten times, and the pressure data from the force sensor were recorded.

#### 2.2.2 Accuracy Tests Under Visual Guidance

**FIGURE 4:**
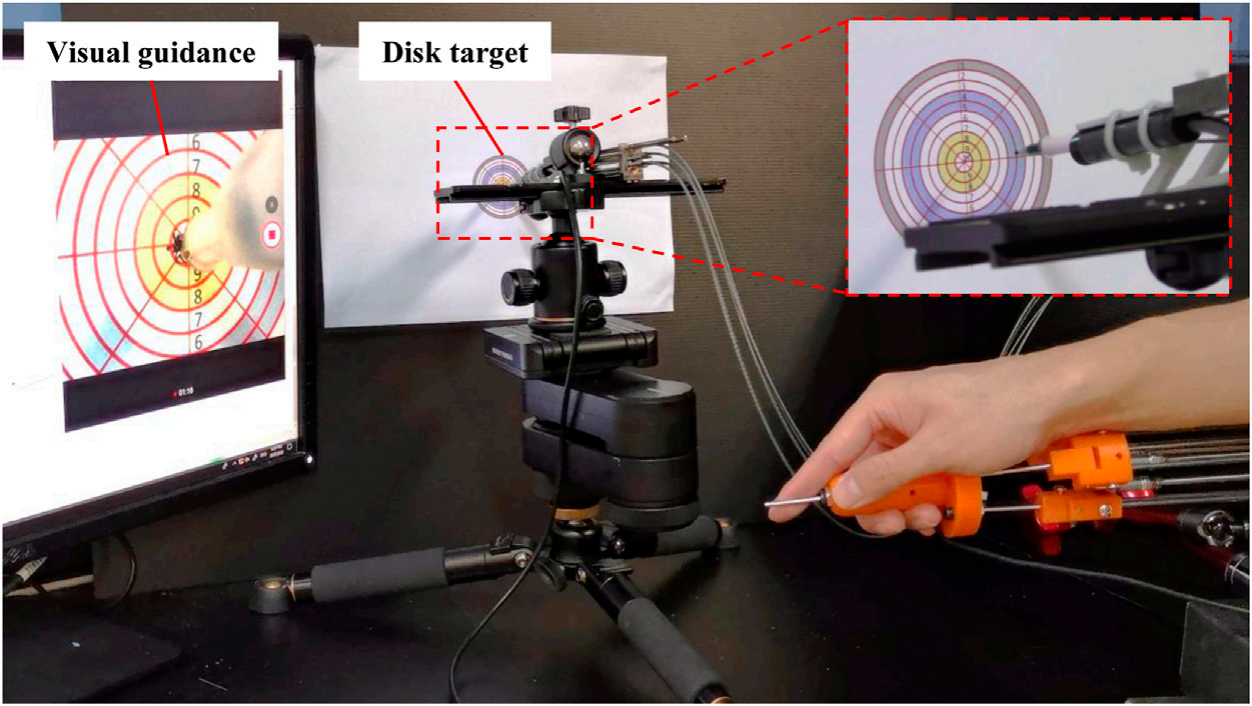
Setup for the accuracy tests of master-slave operation under the visual guidance.

The efficiency of sampling and the validity of the samples are highly related with the operative accuracy, especially for the master-slave operation under visual guidance. The tests of the master-slave operation with following the reciprocating motion and circle trajectory tracking have been conducted in our previous work (Li et al., 2019a; Li et al., 2019b). As a result, the accuracy of the master-slave operation under visual guidance was tested by a simple method as follows. As shown in Figure 4, the disk target with ten concentric and equally distributed circles was designed. The diameters of the first and 10th circles were 50 and 5mm, close to the diameters of the opened mouth and the uvula. A ballpoint pen refill was attached to the terminal of the manipulator instead of the swab stick. The disk target was placed in front of the manipulator with a distance of 20 mm. Under the guidance of the real-time video from the camera, the manipulator was driven to move towards the center of the disk target by operating the master device with an average speed of more than 6 mm/s. This process was repeated ten times.

#### 2.2.3 Performance Tests on the Human Phantom

**FIGURE 5:**
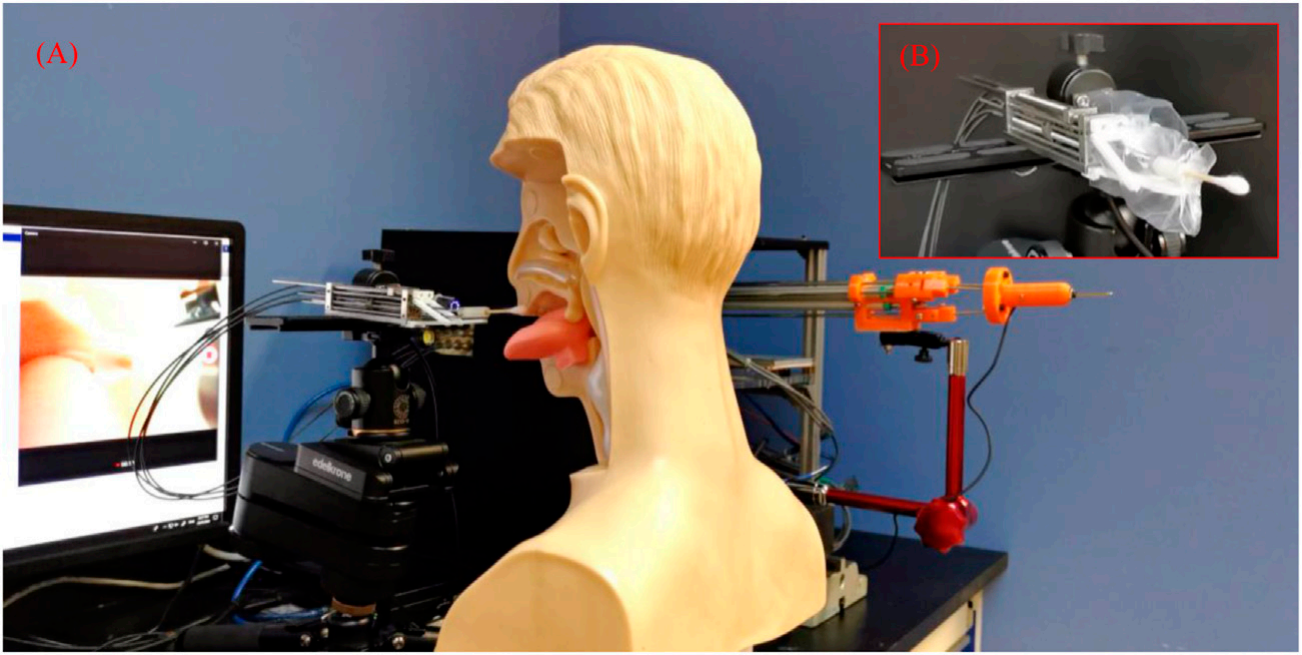
Setup for the performance tests of the robot, **(A)** The setup of the robot, **(B)** The flexible manipulator with sterilized wraps.

The performance of the robot was preliminarily tested on a human phantom to demonstrate the feasibility of sampling. As shown in Figure 5A, the human phantom with a ratio of 1:1 to the human body was placed in front of the robot. The manipulator with a swab stick was controlled by the operator under the visual guidance, aiming to reach the throat and swab it via a swab stick. This process was repeated three times. The method that prevents the robot from being polluted by the virus was shown in Figure 5B. The manipulator was covered with sterilized wraps, and the endoscope was covered with transparent preservative film for a clear vision.

## 3 Results

### 3.1 Pressure of the Tongue Depressor

**FIGURE 6:**
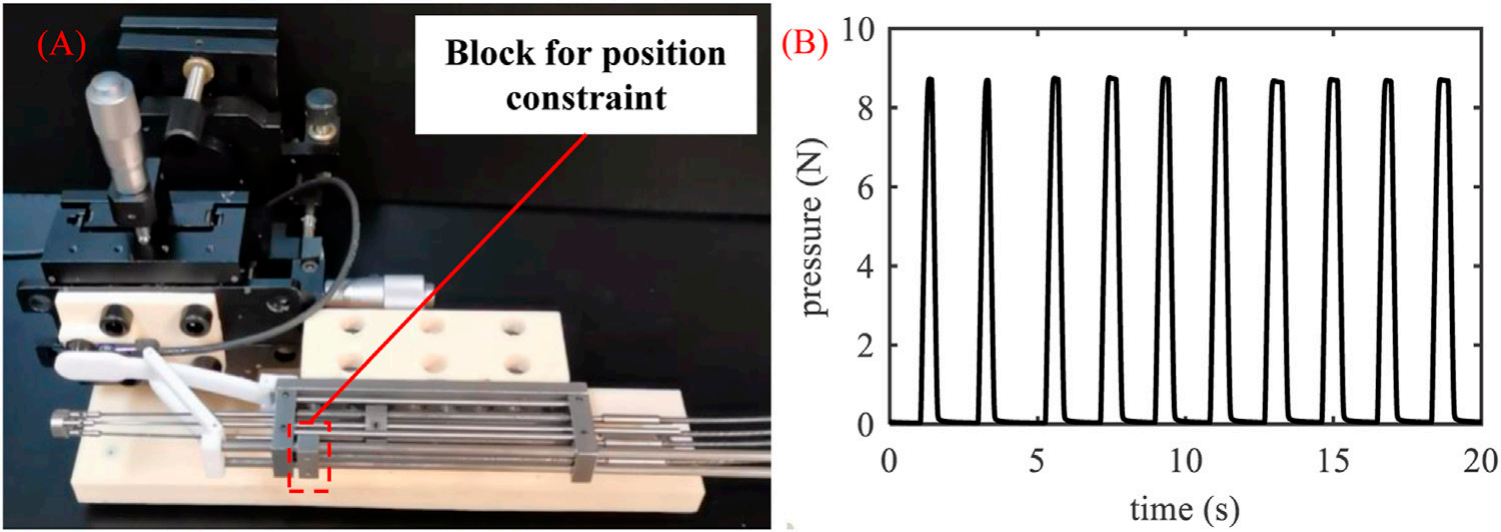
Pressure tests of the tongue depressor, **(A)** Process of the tests, **(B)** The pressure of the tongue depressor with time.

The results of the pressure test of the tongue depressor are shown in Figure 6. Figure 6A shows the state of the tongue depressor when it contacts the force sensor. The deformation of the tongue is caused by the elastic of the polylactic acid (PLA) material. For the safety consideration, the block that drives the tongue depressor is restricted within the manipulator frame to ensure that the rotation of the tongue depressor ranges is in a defined region. Figure 6B shows that the pressure is stable in each test. The average pressure of 10 trials is 8.72 ± 0.03 N. As the pressure is used to prevent the disturbance of the tongue under normal conditions, it should be much larger than the average forward force of the tongue with 2.2 N (Milazzo et al., 2019) in consideration of the individual differences and the stress reaction of the testees. According to the force transmission, the pressure of the tongue depressor is determined by the power of the driving motor, which means it can be further adjusted for practical application if it is not applicable.

### 3.2 Accuracy Under Visual Guidance

**FIGURE 7:**
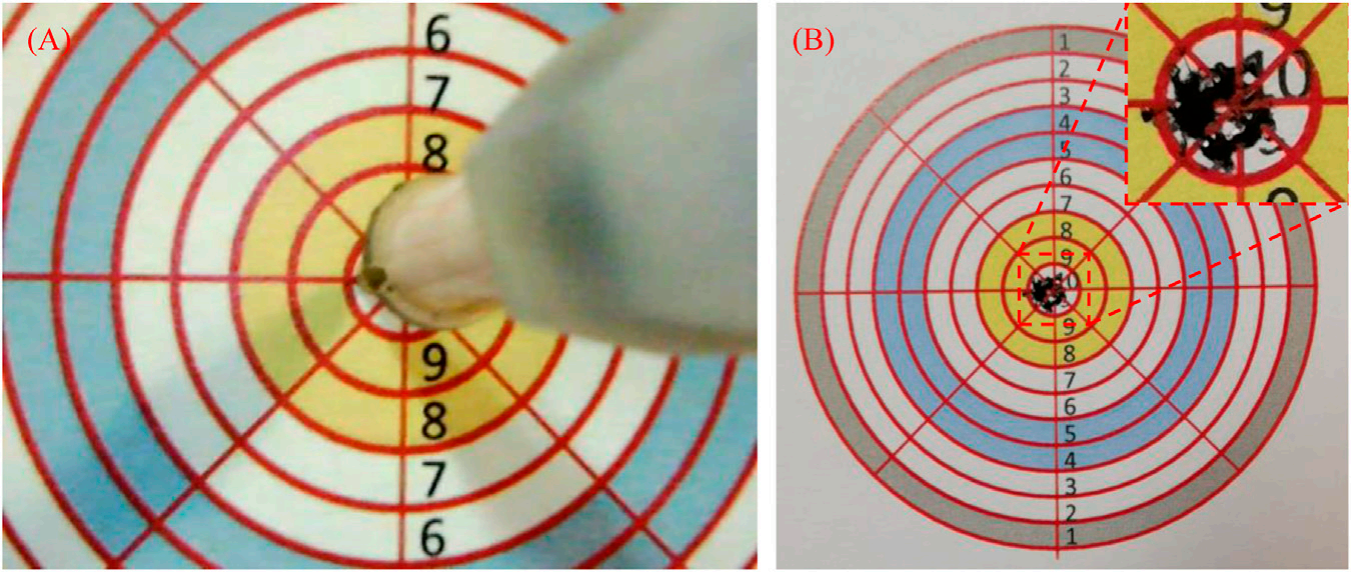
Accuracy tests’ results of master-slave operation under the visual guidance, **(A)** The process of the tests, **(B)** The results.

As shown in Figure 7, all the points drawn by the terminal of the flexible manipulator are located in the 10th circle, which means the accuracy of the manipulator under visual guidance is higher than 2.5 mm during the teleoperation. It is satisfied for sampling because the target region’s diameter is more than 20 mm, which is eight times more than the maximum error. This test is practical and effective as the trial process is close to the sampling operation, though the method is simple without using precision measuring equipment.

### 3.3 Performance on the Human Phantom

**FIGURE 8:**
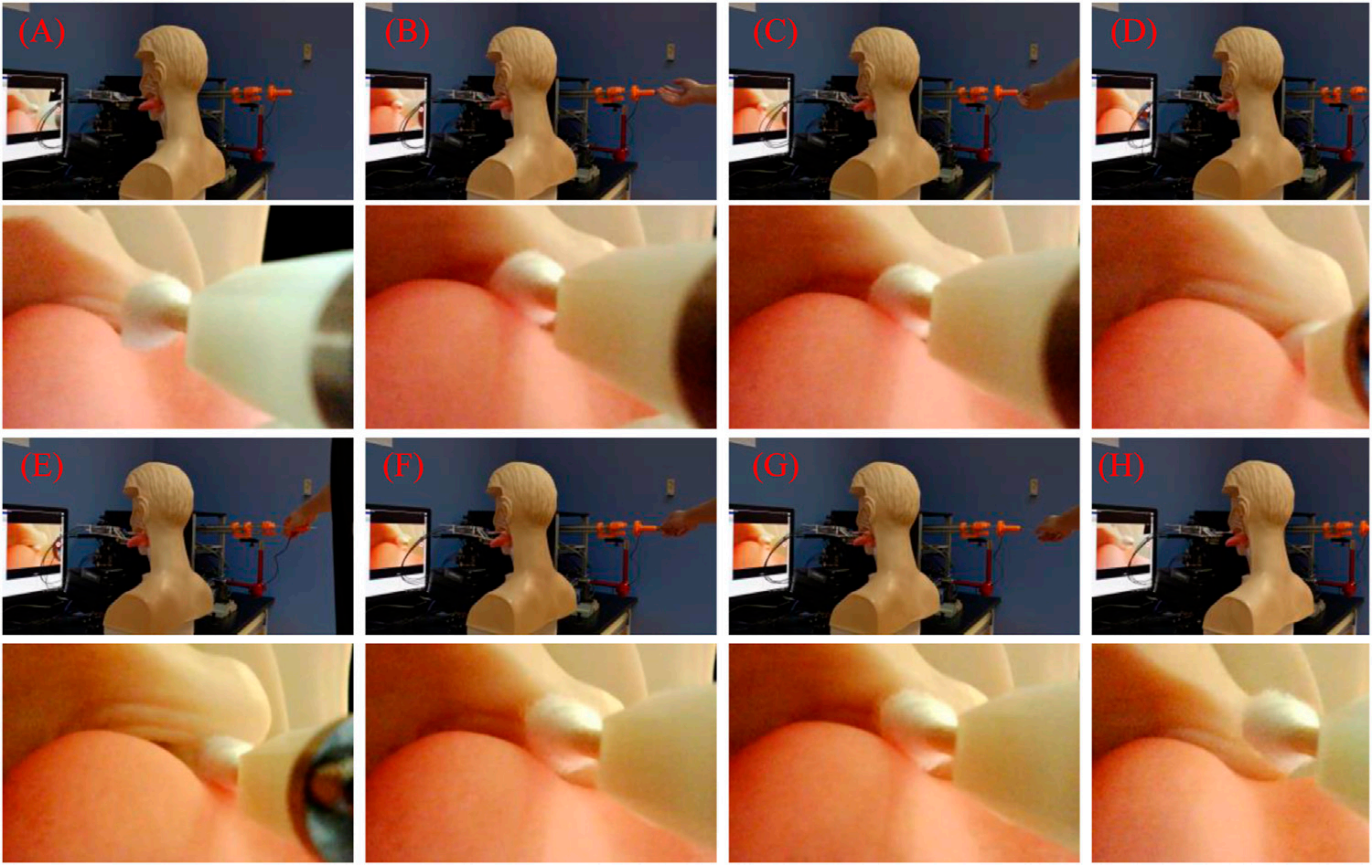
The performance tests’ results of the robot, **(A)** The flexible manipulator was set to the initial state and put on the table, **(B)** The human phantom was pushed forward with the tongue contact to the tongue depressor, **(C)** The tongue depressor was controlled to press the tongue, **(D)** The flexible manipulator was controlled to reach to the target region, **(E)** The flexible manipulator was controlled to get sample via the swab stick, **(F)** The flexible manipulator was controlled to retrieve from the throat, **(G)** The tongue depressor was retrieved, **(H)** The human phantom was pulled back.

As the operation process of the tests is similar each time, we take one as an example, which is shown in Figure 8. The followings are the procedure:

• The flexible manipulator was set to the initial state and put on the table (Figure 8A, time: 3 s).
• The human phantom was pushed forward with the tongue contact to the tongue depressor (Figure 8B, time: 4 s).
• The tongue depressor was controlled to press the tongue (Figure 8C, time: 3 s).
• The flexible manipulator was controlled to reach the target region (Figure 8D, time: 4 s).
• The flexible manipulator was manipulated to get a sample via the swab stick (Figure 8E, time: 6 s).
• The flexible manipulator was controlled to retrieve from the throat (Figure 8F, time: 6 s).
• The tongue depressor was retrieved (Figure 8G, time: 2 s).
• The human phantom was pulled back (Figure 8H, time: 6 s).

After this operation, the operator completed all the procedures, which lasted for 34 s. During the process, c) to g) are conducted under visual guidance. As key steps, the tongue depressor pressed the tongue effectively, and the swab stick was operated dexterously, which is satisfied for the operator. This is only a preliminary test to shown the possible procedure of the operation. More tests on human will be involved in the future to further evaluate the performance of the robot.

## 4 Discussion

A flexible transoral robot with a teleoperated configuration is proposed to address the surgeons’ risks during the face-to-face COVID-19 swab sampling due to the novel coronavirus’s high infectivity. The manipulator with a 3-PU flexible parallel mechanism allows it to achieve proper flexibility, improving testees’ safety. The master device is similar to the manipulator in structure, which is easy to use for operators. Under the guidance of the vision from the endoscope, the surgeon can operate the master device to control the swab’s motion attached to the manipulator for sampling. As a key feature of this robot, the tongue depressor is used because there is a possibility that the tongue blocks the view to swab the back of the throat during the sampling. Hence, the tongue depressor is useful to provide a counterforce. Sterilizability is essential for surgical robotics, especially the sampling robot. Cross infection to testee should be avoided. The common methods, such as sterilized wraps, ultrasonic washing, and pasteurization, are applicable for this robot. As a quick and easy solution, we cover the sterilized wraps manually to the robot. The terminal compliance is combined by the swab with long and thin shaft and the manipulator. As the manipulator is operated under visual guidance, the deformation of the swab can be observed in real-time that indicates the contact force between the swab and the tissue as the safety assurance passively.

In this paper, the robotic system, the workspace, and the operation procedure are described in detail. The experiments are conducted to demonstrate the preliminary feasibility of the robot. The pressure tests of the tongue depressor show that proper pressure can be provided. With visual guidance, the flexible manipulator’s accuracy under the master-slave configuration is acceptable for the sampling operation. The performance tests on the human phantom show the basic operation procedure, providing a reference for application. In practical application, the operator can stand far away from the slave manipulator by extend the cables connecting the controlled and the computer to avoid the face to face sampling. Besides, this robot can also be used for self-testing. The master-slave configuration under visual guidance makes the test easier. And the disturbance of the tongue can be reduced by the tongue depressor.

As a surgical robot, many difficulties need to be overcome before its clinical deployments. More experiments will be conducted to evaluate the performance of the robot, such as the success rate of sampling and the testee’s adverse reaction. The fully autonomous operation will be taken into consideration.

## Data Availability

The original contributions presented in the study are included in the article/Supplementary Material, further inquiries can be directed to the corresponding author.
